# Deep sequencing of microRNAs reveals circadian-dependent microRNA expression in the eyestalks of the Chinese mitten crab *Eriocheir sinensis*

**DOI:** 10.1038/s41598-023-32277-1

**Published:** 2023-03-31

**Authors:** Changyue Yu, Zhiwei Huang, Yingkai Xu, Baoli Zhang, Yingdong Li

**Affiliations:** Key Laboratory of Livestock Infectious Diseases in Northeast China, Ministry of Education, College of Animal Science and Veterinary Medicine, Shenyang Agricultural University, Shenyang, 110866 China

**Keywords:** Biotechnology, Endocrinology

## Abstract

MicroRNAs (miRNAs) are small endogenous non-coding RNAs. In crustaceans, miRNAs might be involved in the regulation of circadian rhythms. Many physiological functions of crustaceans including immunity and hormone secretion exhibit circadian rhythms, but it remains unclear whether specific miRNAs contribute to the alteration of crustacean physiological processes under circadian rhythms. This study investigated the mechanisms of miRNA regulation of circadian rhythms in the Chinese mitten crab (*Eriocheir sinensis*), one of China's most important aquaculture species. We obtained eyestalks from crab specimens at four time points (6:00; 12:00; 18:00; 24:00) during a 24-h period. We identified 725 mature miRNAs, with 23 known miRNAs differentially expressed depending on the time of day. The Gene Ontology (GO) and Kyoto Encyclopedia of Genes and Genomes (KEGG) pathway enrichment analyses revealed that the putative target genes for differentially expressed miRNAs were significantly enriched in the immune response and endocrine-related pathways. Numerous putative target genes are involved in the circadian-related pathways and enriched on circadian-control genes. These results suggest that the expression of miRNAs regulates some specific physiological functions in *E. sinensis* under circadian cycles. We also profiled various putative target genes enriched under the circadian-related pathway. This study performed miRNA expression in the eyestalks of *E. sinensis* during a 24-h daily cycle, providing insights into the molecular mechanism underlying crustacean circadian rhythms and suggesting miRNAs' role in studying crustacean physiology should not be overlooked.

## Introduction

Small RNAs (sRNAs), ranging from 18 to 24 nucleotides (nt) in length, are key post-transcriptional regulators of gene expression in many eukaryotes. One unique class of sRNAs, known as microRNAs (miRNAs), is an endogenous class of sRNAs of about 20–24 nt in length. miRNAs are essential in animal and plant physiological processes^1–3^. In crustaceans, many evolutionarily conserved miRNAs have been identified with specific expression patterns that regulate various physiological and behavioral processes, including reproduction^4,5^, immunity^6^, growth^7^, apoptosis^8^, and circadian^9^.

Across the animal kingdom, circadian rhythms have an approximate 24-h oscillation, which appears to confer a selective advantage by timing biological processes to anticipate the transitions between day and night^10^. Exogenous, stimulus-sensitive input pathways, a central clock pacemaker capable of coordinating different endogenous pacemakers, and output pathways that produce overt rhythms comprise the clock's mechanism in invertebrates. *Drosophila*, as a fundamental model of invertebrates, has been extensively researched for miRNA roles in the regulation of the circadian clock system. MiRNA participates in rhythm regulation by interacting with genes of the clock system, like the expression level of *timeless* could be regulated by mir-276a^11^, which could strengthen *Drosophila* circadian rhythms, and miR-375 could modulate the circadian rhythm and sleep via targeting *timeless*^12^, and Let-7 regulates the circadian rhythm via repression of *Clockwork Orange*^13^. In invertebrates, a tiny number of miRNAs have strong clock-regulated rhythms, and miRNA-mediated post-transcriptional regulation modulates daily fluctuations in protein levels for select proteins. MiRNAs are expected to act in rhythmic expression by providing a fine-tuning mechanism that collaborates with transcriptional and post-translational pathways to form an oscillatory system that can alter the levels of individual proteins to values optimal for a certain time of day.

The eyes are the primary visual organs in all animals, responsible not only for motion and color (picture) perception but also for transferring light information to the brain's circadian clock. The synchronization of circadian clocks with the external environment (also known as circadian entrainment) is necessary for their adaptive function and serves as a critical link between the clocks and the environment^14^. Circadian entrainment is a difficult task, as evidenced by a large number of photoreceptor pigments and organs involved. However, the way light entrains the circadian clock appears to differ between animals. Most arthropods utilize their eyes, but they also use specialized photoreceptors to communicate information about environmental light conditions to the circadian system. *Drosophila* is unique in that it expresses the blue light-sensitive cryptochrome (CRY) directly in its circadian clock neurons, and CRY is commonly thought to be the fly's primary circadian photoreceptor^15^. Photoreceptors in the eyes and the body wall of *Drosophila* cooperate in mediating time-dependent color preference and light avoidance^16^. In crustaceans, the eyestalk functions as a central pacemaker that transmits light and dark signals^17^, making this a critical organ for crustacean circadian rhythm research. Notably, the eyestalk can also secrete specific, life-sustaining hormones, which may be directly responsible for the continued life of the crustacean. Red pigment concentrating hormone (RPCH), the pigment dispersing hormone (PDH), and the different members of the crustacean hyperglycemic hormone, molt-inhibiting and gonad-inhibiting hormone family (CHH/MIH/ GIH peptide family) are responsible for the regulation of immunity, growth, molt, and reproduction in crustaceans. Hence, crustacean neuroendocrine studies often target eyestalk. Back in 1995, a study used in situ hybridization, northern blotting, and RNase protection assays showed that mRNAs encoding crustacean endocrine hormones were expressed in the eyestalk^18^. Additionally, the previous recording of the electrical response of crayfish to light by electroretinography revealed a high-frequency circadian cycle, suggesting that calcium-dependent signals can enhance the coupling mechanism of circadian signals in the crayfish eyestalk^18^. Another study on fiddler crabs found diurnal variations in the red pigment dispersing hormone (RPDH) levels and 5-hydroxytryptamine (5-HT) in its eyestalk^19^. However, studies that focus on investigating crustacean circadian rhythms at the gene and miRNA levels are far from hearing. Besides, there is considerable evidence that systemic cues and molecular clocks within immune cells directly control the immune system's circadian rhythm and that autonomic and endocrine outputs of the circadian system communicate time-of-day information to immune tissues^20^. Clock-controlled genes and immune function-related transcription factors are involved in innate and adaptive immune responses^21^. Several studies have reported associations between circadian rhythm and pathogenesis^22,23^. However, most mechanism studies on the effects of circadian rhythms on animal immune function have relied on specific mammalian and arthropod models. Notably, there is no study indicates that crustacean miRNA is involved in the regulation of physiological systems governed by its circadian rhythm, such as immunological and endocrine functions.

As an aquacultural economic product, the Chinese mitten crab (*Eriocheir sinensis*), a decapod crustacean, supports traditional freshwater fishery. It is widely distributed in China and is an invasive species in Europe and the United States^24^. The crab is valued for its flavor and is sold in domestic and international markets^25^. Furthermore, a previous study on *E. sinensis* identified five molting genes differentially expressed in the eyestalk over a 24-h period; specifically, the expression patterns suggested that the crab molts at night, similar to another crustacean^26^. Our recent study on the hemolymph transcriptome of crustaceans has further shown that crustaceans exhibit significant changes in immune response and digestive metabolism during the 24-h circadian cycle^27^. Although miRNA regulation is critical to circadian rhythms, no studies have investigated miRNA expression profiles in crustaceans under circadian rhythm^28^. As a result, RNA sequencing (RNA-seq) was used in this study to analyze the miRNA expression profiles in the eyestalk of *E. sinensis*. We determined the miRNA distribution at four time points throughout 24 h and identified any differentially expressed miRNAs based on the time of day. The present study reported the profile of crab eyestalk miRNA, providing helpful insight into the molecular basis of crustacean circadian rhythms. Such information might help improve crab breeding programs using RNA-based molecular technology.

## Materials and methods

### Material preparation and total RNA isolation

In December 2020, healthy *E. sinensis* individuals (n = 100; average weight: 18.62 ± 4.49 g) were collected from a wintering pond in Panjin City, Liaoning Province, China, and moved to the Aquaculture Laboratory of Shenyang Agricultural University. The crabs were placed in two 300-L tanks with a recirculating tap water system. Each tank was continuously supplied with oxygen and maintained at 15 ± 5 °C and a 12:12 h light/dark cycle. The crabs were temporarily reared for two weeks on an artificially formulated diet, and they were fed once a day at 16:00, with the feed accounting for 5% of the total body weight of each group, and the last feeding time was 48 h before the experiment began. Totally 80 healthy crabs were randomly divided into four groups without considering their sex. The entire experiment was carried out at four time points within 24 h, each with a 6 h interval sampling, and at each time, 18 crabs' eyestalks were taken and randomly placed in three 2-mL sterile centrifuge tubes, each having six crabs' eyestalks (the sex of crabs was also randomly selected). The four time points are 06:00, 12:00, 18:00, and 24:00. The first two time points were daytime and sampling was conducted under natural light. The sampling occurred during the winter in northeast China (dark by 18:00); therefore, the last two time points were in the dark, with samples collected under black and red light to avoid the influence of external light sources. Eyestalk samples were stored at − 80 °C until DNA extraction.

Total RNA was extracted from eyestalk samples using the TRIzol reagent (Invitrogen, Carlsbad, CA, USA); the degradation and contamination were monitored on 1% agarose gels, after which the quantity and purity of RNA were assessed using a Nanodrop 8000 spectrophotometer (Nanodrop Technologies, Wilmington, DE, USA). The RNA Integrity Number (RIN) was verified on an Agilent 2100 Bioanalyzer (Agilent Technologies, Santa Clara, CA, USA).

### sRNA library construction and sequencing

The sRNA libraries were constructed using the NEBNext Multiplex Small RNA Library Prep Set for Illumina (New England Biolabs, Ipswich, MA, USA) according to the manufacturer's guidelines and protocol. Briefly, 1 μg of RNA was ligated to 3′ and 5′ adapters using the ligation enzyme mix, then reverse-transcribed using Superscript II reverse transcriptase. Amplification was executed to obtain the PCR products. sRNA libraries were analyzed as part of the quality control, and the average size of inserts was approximately 140–150 bp. The sequencing library was quantified using the Agilent high-sensitivity DNA assay on a Bioanalyzer 2100 system (Agilent). The library was then sequenced on NovaSeq 6000 platform (Illumina) by Shanghai Personal Biotechnology Co., Ltd. (Shanghai, China). Raw data presented in this paper have been deposited in the NCBI Short Read Archive (http://www.ncbi.nlm.nih.gov/sra/) and are accessible through the accession number SRR15367803.

Clean reads with sequence lengths greater than 18 nt and less than 36 nt were screened; high-repeated reads were also removed. The annotation of the sRNA sequences was organized according to the known miRNA > piRNA > rRNA > tRNA > snRNA > snoRNA > novel miRNA priority to ensure that each sRNA has a unique annotation. Since mature miRNA information of *E. sinensis* was not available in miRBase 22, any remaining reads not annotated to the four ncRNAs were BLASTed against mature miRNA sequences of all animals data in miRBase22 (http://www.mirbase.org/)^29^, with E-value threshold of 1e−5. Sequences not annotated with any information used an integrated analysis of Mireap (V2.0)^30^ and miRDeep2^31^ for new miRNA prediction analysis.

### Differential expression and functional analysis

Known miRNAs were identified at four points within 24 h (06:00 vs. 12:00, 06:00 vs. 18:00, 06:00 vs. 24:00, 12:00 vs. 18:00, 12:00 vs. 24:00, and 18:00 vs. 24:00) were analyzed using DESeq (V 1.18.0)^32^. Differential expression was determined according to the criteria of | fold change |> 2 and adjusted *p*-value < 0.05. The Pheatmap package (V1.0.10) was used for the bidirectional clustering analysis and heatmap plotting of genes and samples^33^. The process of clustering among data based on the Euclidean distance^34^ and longest distance (complete linkage)^35^. Potential target genes for differentially expressed miRNAs were predicted using two bioinformatic algorithms, TargetScan (http://www.targetscan.org/) and miRanda (http://www.microrna.org/microrna/home.do)^36^. The putative target genes were then subjected to functional enrichment analysis using the Gene Ontology (GO, http://www.Geneontology.org/) and Kyoto Encyclopedia of Genes and Genomes (KEGG) pathway, http://www.genome.jp/kegg/ko.html) databases. Hypergeometric distribution was used to calculate the term with significant enrichment of target genes, with the *p*-value (FDR) < 0.05.

### Reverse transcription-quantitative PCR (RT-qPCR) validation

Real-time quantification of miRNAs can verify the accuracy of our sequencing results. Total RNA was isolated from the same samples used for sRNA sequencing using a miRcute miRNA isolation kit and then reverse-transcribed using the miRcute Plus miRNA First-Strand cDNA kit (TIANGEN Biochemical Technology (Beijing) Co., Ltd, Beijing, China). The U6 snRNA gene was employed as an endogenous control. Quantitative real-time PCR was performed using the SYBR Premix Ex Taq kit (Takara Bio Inc., Shiga, Japan) and Bio-Rad IQ™5 system (Bio-Rad, Hercules, CA, USA). Primer sequences used for miRNA qRT-PCR are listed in Table S1. We also validated gene expression of the rhythm functional genes of microRNA regulatory targets, four clock system genes (Clock, Cryptochrome, Period, Brain, and muscle Arnt-like protein-1) were selected and determined using quantitative reverse transcription-polymerase chain reaction (qRT-PCR). The cDNA templates from the four time points groups were reverse-transcribed using the Prime Script RT reagent Kit. The SYBR Green RT-PCR assay was performed using an ABI PRISM 7300 Sequence Detection System (Applied Biosystems). β-actin was used as an internal reference. Primer sequences used for mRNA qRT-PCR are listed in Table S2. All the primers in this section were synthesized by Shanghai Sangon Biotechnology Co. Ltd. (Shanghai, China). The 2^−ΔΔCt^ method was used to measure the relative expression of genes. The results were analyzed by one-way ANOVA in SPSS 22.0, with a significance set at *p*-value < 0.05.

### Ethics

The protocols for the sample collection of crabs were approved by the Ethics Committee of Shenyang Agriculture University.

## Results

### Global analysis of sRNA sequencing data

Via the eyestalks miRNA-sequencing, we generated 30,421,570 raw reads from the 06:00 samples; 34,702,269 from the 12:00 samples; 41,471,050 from the 18:00 samples; and 29,510,425 from the 24:00 samples. After discarding low-quality reads and adaptor sequences, we obtained 24,548,555 clean reads greater than 18 nt in length. Samples collected at 18:00 yielded the highest number of raw and clean reads. For raw data and filtered sequencing results per sample, see Table S3.

We plotted the sequence length distribution for both total and unique reads in eyestalk samples from the four points across 24 h (Fig. S1). sRNAs ranged from 18 to 36 nt and exhibited a distinct single peak distribution at 22 nt. In addition, sRNA less than 24 nt were dominant, indicating that *E. sinensis* eyestalks mainly expressed the short-size class.

### Identification of miRNAs in the eyestalk

**Figure 1:**
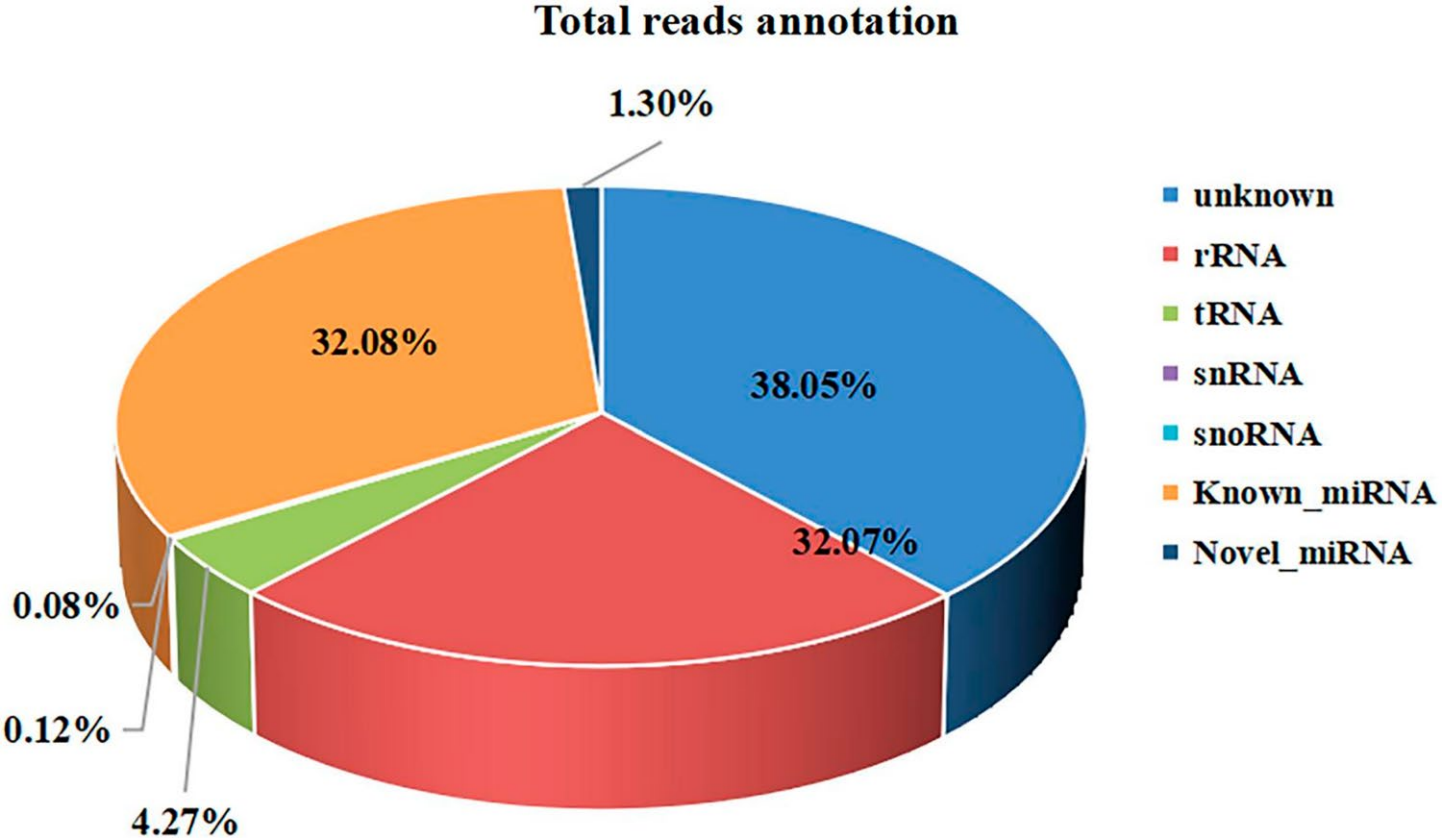
Distribution of small RNAs in the eyestalk. (**a**) The eyestalk of *E. sinensis.* (**b**) Total reads annotation classification.

We identified 725 mature miRNAs expressed across the four points (Table S4), with 522 miRNAs at 6:00, 620 at 12:00, 697 at 18:00, and 581 at 24:00 (Table 1), and 474 known miRNAs were universally expressed. Our present study identified 147 novel miRNAs (Table 2). Mature miRNAs were the most abundant, accounting for 33.38% of total reads collected during the 24-h period (Fig. 1).

**Table 1:** The results of the known miRNA sequences.

Group	miRNA	Unique reads	Total reads
6:00	552	7222	18,827,440
12:00	620	7990	23,635,798
18:00	697	8660	28,569,838
24:00	581	7638	23,454,934

**Table 2:** The statistical results of novel miRNA annotation.

Group	Novel miRNA	Unique reads	Total reads
6:00	126	2507	859,340
12:00	127	2781	967,638
18:00	140	2839	1,123,581
24:00	120	2672	872,724

### Differential expression of miRNAs

We found 14 and 17 differentially expressed miRNAs in the 06:00 vs. 12:00 and 12:00 vs. 24:00 groups, respectively (Table 3). The number increased to 19 and 27 for the 06:00 vs. 24:00 and 12:00 vs. 18:00 groups, respectively. The 06:00 vs. 18:00 group yielded a much higher number of differentially expressed miRNAs (64); 61 were upregulated, and 3 were downregulated. Notably, 54 differentially expressed miRNAs were downregulated in the 18:00 vs. 24:00 group. None of the differentially expressed miRNAs were upregulated in this comparative group. Interestingly, the pairwise comparison groups containing the 18:00 group both produced significantly higher numbers of differentially expressed miRNAs, and in subsequent functional enrichment analysis, we also found significant differences between the miRNAs identified at 18:00 and those identified at the other three time points.

**Table 3:** The statistical results of expression difference analysis.

Control	Case	Up-regulated	Down-regulated	Total
6:00	12:00	14	0	14
6:00	18:00	61	3	64
6:00	24:00	11	8	19
12:00	24:00	2	15	17
18:00	24:00	0	54	54
12:00	18:00	26	1	27

**Figure 2:**
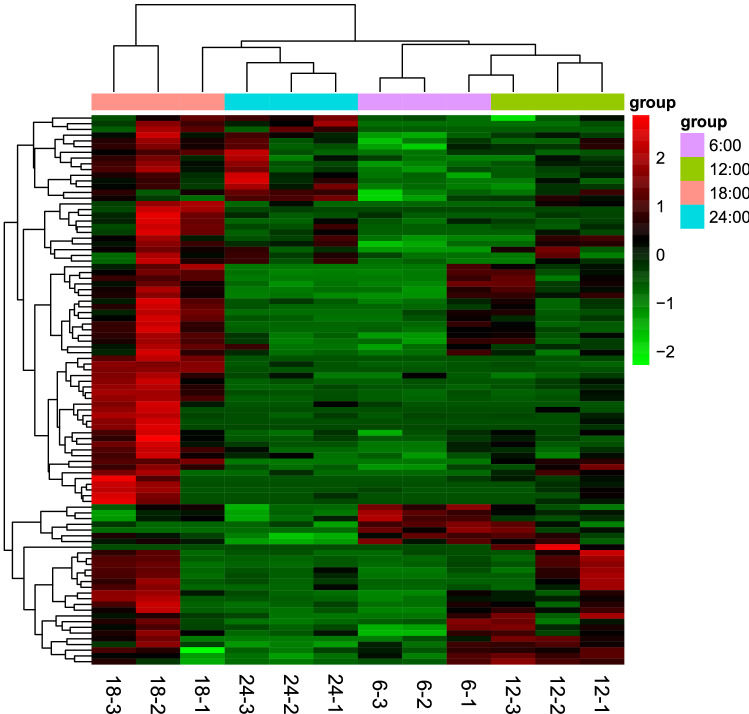
Cluster heatmap of the miRNAs. Each row represent one differentially expressed miRNA.

**Figure 3:**
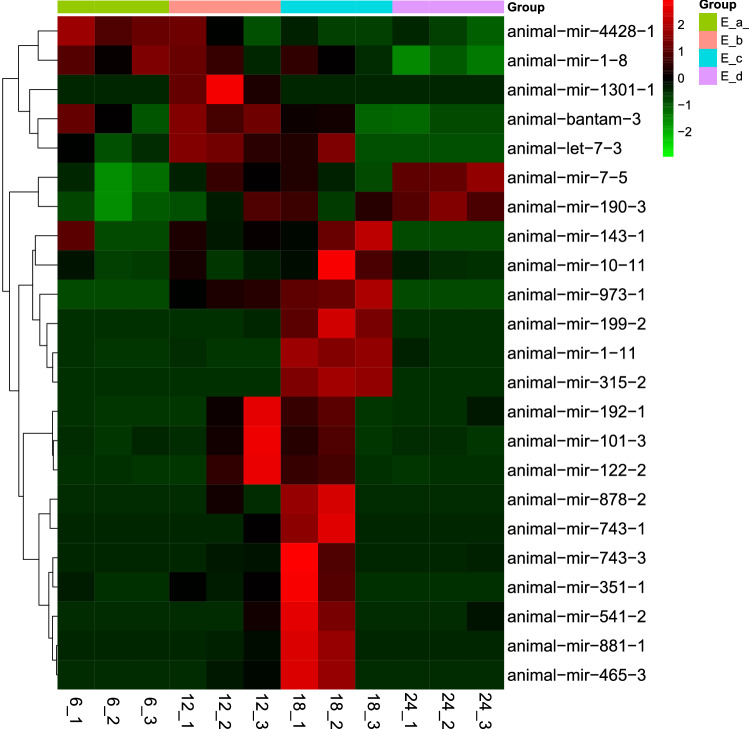
Cluster analysis of expression trend of differentially expressed miRNAs.

We then clustered DE miRNAs showing the same or similar expression patterns. Eyestalk samples from 18:00 showed significantly higher miRNA expression than samples from the other three time points (Fig. 2). Finally, we obtained 23 differentially expressed known miRNAs. Furthermore, most of the known miRNAs that appeared to be differentially expressed in the present study showed peak expression at 18:00 (Fig. 3).

### Prediction and functional analysis of miRNA target genes

We predicted 90,748 target genes and 583,972 target sites for the differentially expressed miRNAs in *E. sinensis* eyestalks. We focused on functionally annotating the target genes of miRNAs from 18:00 because this time point yielded the highest number of differentially expressed miRNAs, with significantly greater expression than the other time points. GO analysis demonstrated that target genes were mainly involved in immune functions for all groups, including regulation of transferase activity, hydrolase activity, catalytic activity, and oxidoreductase activity (Fig. S2). Endopeptidase activity was the most enriched pathway in the 18:00 vs. 24:00 group.

**Figure 4:**
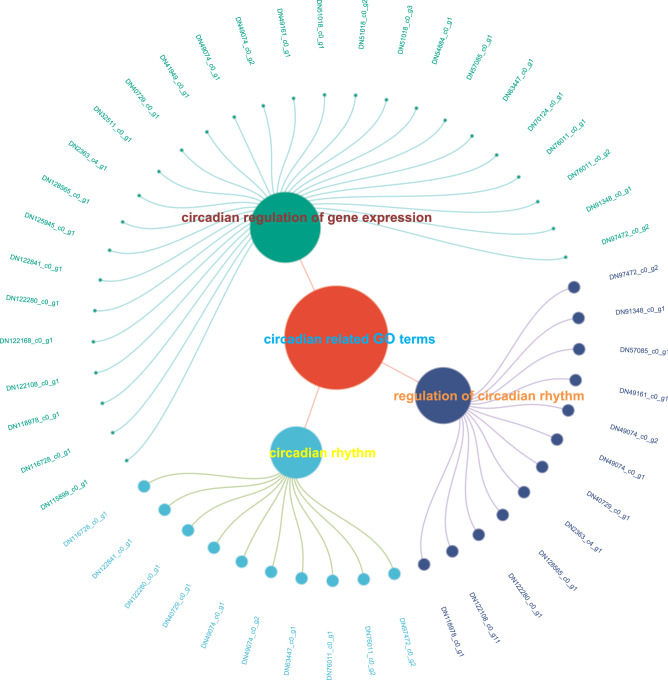
Enrichment of putative target genes on circadian-related GO terms. The size of the circles of the first level (all circadian-related terms) is based on the number of genes contained under different terms, and the size of the circle of each gene is based on the value of the target gene expressed in the term.

The GO enrichment analysis results integrating all target genes showed putative target genes of differentially expressed miRNAs that might primarily be associated with transferase activity and catalytic activity in crabs according to the molecular function analysis (Fig. S3). Notably, putative target genes were highly valued with enrichment rates in three circadian-related GO terms. Of the 29 genes involved in the term "circadian rhythm," we obtained 27 hits; "regulation of circadian rhythm" received 12 hits out of 14 genes; and "circadian regulation of gene expression" was listed in 10 out of 12 genes (Fig. 4). KEGG enrichment analysis revealed that putative target genes were mainly enriched in the pathways related to the endocrine system and metabolism (Table S5). Moreover, critical components in the CLOCK system such as circadian entrainment, and MARK singaling enriched with some circadian-related genes like CRY, PER, and BAML 1. These components were involved in the circadian rhythm pathway which is enriched with abundant putative target genes (Fig. S4).

### Validation with RT-qPCR

**Figure 5:**
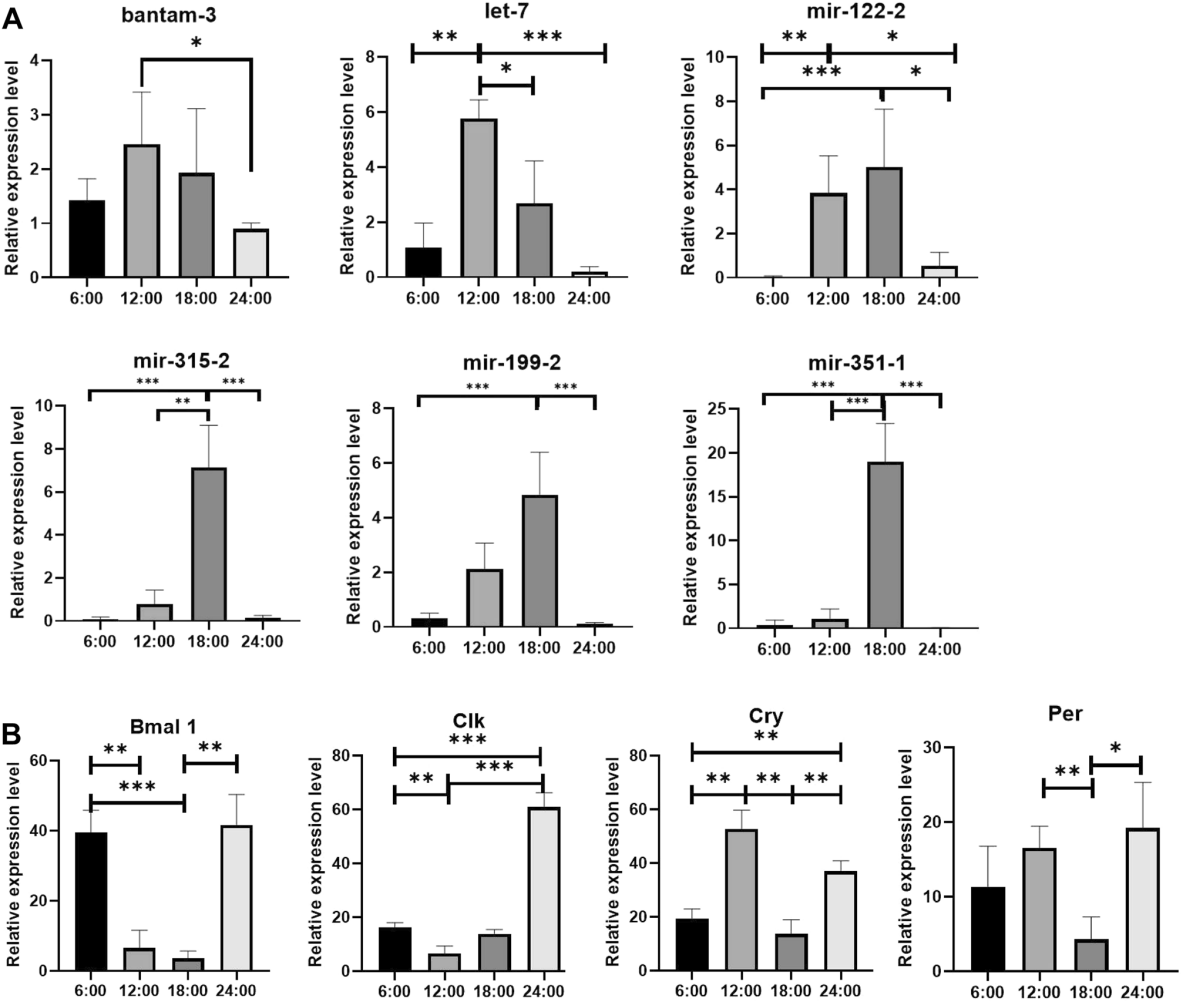
RT-qPCR validation of the differentially expressed miRNAs. (**A** Expression levels of six differentially expressed miRNAs normalized to the expression level of U6 of *E. sinensis* with different treatments.) (B Expression levels of four target CLOCK system genes normalized to the expression level of β-actin of *E. sinensis* with different treatments.) Groups marked "*" represented a significant difference at P < 0.05, "**" represented a significant difference at P < 0.01, "***" represented a significant difference at P < 0.001. Statistical analyses were performed using a one-way ANOVA test in GraphPad Prism (V 8.0).

Among the 23 differentially expressed known miRNAs, we selected 6 miRNAs randomly for RT-qPCR validation of the miRNA-seq data (Fig. 5A). The expression level of four rhythm functional genes of miRNA regulatory targets in the COLCK system was also performed in RT-qPCR validation (Fig. 5B).

## Discussion

In the current investigation, we characterized the initial miRNA transcriptomic profile in crustaceans under daily rhythms. Through the identification of 725 mature miRNAs, we observed that the majority of differentially expressed miRNAs exhibited peak expression at 18:00 under circadian conditions. Notably, the putative target genes of differentially expressed miRNAs were found to be enriched in pathways related to immune and endocrine processes, and many target genes were also enriched in terms associated with circadian regulation. Despite a well-established understanding of the role of some miRNAs in circadian rhythm in the invertebrate model *Drosophila*, research on this topic in crustaceans remains limited. This study represents the first identification of the miRNA profile of the eyestalk of *E. sinensis* under the 24-h day cycle. The findings of this study contribute to the understanding of miRNA identification under circadian rhythms in crustaceans, providing primary molecular data in this field.

The transcriptional profile of the eyestalk tissue of the crustacean species *E. sinensis* was examined for the presence of microRNAs (miRNAs) associated with circadian rhythm regulation in this study. Previous research has identified miRNAs such as miR-219 and miR-132 in the mammalian brain as regulators of circadian rhythm via different pathways^37^. However, in the present study, while miR-219 and miR-132 were identified in the *E. sinensis* eyestalks, there was no evidence of a circadian-dependent difference in their expression. Similarly, miR-263, which has been shown to exhibit strong diurnal variation in wild-type *Drosophila*^38^, was found in the miRNA transcriptome of the eyestalk but did not display a time-dependent expression pattern. Despite the presence of circadian rhythms in mammals, fruit flies, and crustaceans, the tissues and organs that regulate these rhythms vary among different species. In our study, we focused specifically on the eyestalk tissue of crustaceans. Numerous putative target genes of differentially expressed genes were enriched on circadian-related terms. Especially the components of the circadian clock system like CREB1, BMAL1, CRY, PER, and CLOCK enriched with most of the putative target genes, indicating the potential miRNA regulation mechanism in *E. sinensis*. These components are mainly concentrated in the downstream part of the circadian pathway and are involved in the regulation of clock output, suggesting that the differentially expressed miRNAs of circadian rhythms in *E. sinensis* may be mainly involved in the regulation of the clock outputs biological processes of this species. We further profiled the target genes annotated to circadian-related GO terms. Since the target genes under all three terms associated with circadian rhythms had high hit rates, this might provide further evidence that eyestalk-miRNAs play a regulatory function on circadian rhythmicity in crustaceans. However, given the lack of information on the specific tissue sites of circadian rhythm regulation in *E. sinensis*, we cannot currently identify any miRNAs that exhibit fluctuating levels under circadian rhythms in other neural tissues of this species. Further research in this area is necessary to fully understand the relationship between miRNAs and circadian rhythms in this species. Notably, our results indicate that miR-122, a miRNA associated with circadian rhythms, is significantly differentially expressed in our study. This is consistent with previous research on mammals, suggesting that certain miRNAs, such as miR-122, may play a critical role in regulating the circadian rhythms of crustaceans^39,40^. In recent studies, the role of *bantam* family members in circadian rhythmicity in *Drosophila* has been investigated. Behavioral and molecular experiments have demonstrated that the developmental regulator *bantam* plays a key role in the core circadian pacemaker. Cellular biochemical experiments have shown that bantam can regulate CLK translation through association with three target sites within the CLK untranslated region (UTR)^41^. Furthermore, it has been observed that the expression level of miR-*bantam* exhibits diurnal variation in crustaceans for the first time. However, it is worth noting that other differentially expressed miRNAs have not been identified as regulators of circadian rhythms in invertebrate models. This discrepancy may be attributed to interspecific differences and variations across the tissues studied, as well as the possibility that some miRNAs may not exhibit daily fluctuations but could still play an important role in circadian regulation. Additionally, the use of the miRBase database as an annotation reference may not be entirely accurate as it may miss genuine miRNAs filtered out by strict annotation criteria and may result in false-positive annotations^42^. To mitigate this issue, future publication of miRNA data resources on crustaceans may improve the accuracy of miRNA screening. It is also important to note that in non-model organisms, studies often assume miRNA-target recognition rules that may not reflect the organism's biology under study^43,44^. Therefore, it is crucial to use an approach such as the intersection results from TargetScan and miRanda to predict potential target genes, as this method aims to reduce the false-positive rate of target prediction and circumvent the limitations of using a single software^45,46^.

According to the GO and KEGG enrichment analysis, most putative target genes of differentially expressed miRNAs are abundantly enriched in the pathways involved in the endocrine and immune systems. One of the most important functions of circadian rhythms is the regulation of the immune system, and the relationship between circadian rhythms and the immune system is complex^47^. The most important functions of the circadian rhythm of the immune system are the regulation of the inflammatory response and the immune response to infections. Cell adhesion molecules and antioxidative enzymes in a crustacean possibly play role in immunity, previous research on *E. sinensis* has revealed that the activity of antioxidant enzymes, such as malondialdehyde, superoxide dismutase, glutathione peroxidase, and catalase, fluctuates in different tissues (muscle, hemolymph, and gill) depending on the time of day^48^. This circadian activity of antioxidant enzymes may explain the significant upregulation of miRNAs that we observed at 18:00. Another study showed that molt and immune-related genes were significantly differentially expressed in *E. sinensis* hemolymph collected at 18:00 compared to 6:00, which suggests the molting and immunity of *E. sinensis* showed significant change in daily circadian rhythm^27^. Furthermore, miR-7, one of the differentially expressed miRNAs we identified, plays a positive role in regulating immunity during host-virus interactions^49^. MiR-1, miR-let-7, miR-7, miR-190, and miR-143, are known to be immune-related miRNAs associated with the immune response against pathogens in *E. sinensis*^50^. These miRNAs appeared to be significantly differentially expressed under circadian rhythms in our study, and most of the putative target genes were enriched in immune-related pathways, which also corroborated the strong correlation between the circadian rhythms of crabs and their immune functions. Previous studies have shown that miRNA expression may be positively correlated with or contrary to corresponding gene expression trends^51^. Combining these data suggest that crab immune response could vary under daily rhythm, possibly upregulated around 18:00, which is consistent with recent studies in our laboratory where diurnal variation in the gut bacterial community of *E. sinensis* showed that its immune function was more robust at 18:00^52^. Furthermore, we found endopeptidase and catalytic pathways enriched with a large number of putative target genes under circadian. Endopeptidase is closely related to crustacean endocrine and immunity^53,54^. In rat brains, endopeptidase activity exhibits a precise circadian rhythm in the whole brain and other regions of the central nervous system^55^. There is no evidence that miRNA can regulate endopeptidase circadian rhythms in invertebrates. However, as a part of the nervous system of crustaceans, the eyestalk functions similarly to the mammalian brain tissue. Therefore, crabs may regulate eyestalk miRNA and secrete peptides at 18:00, the hour of transition from day to night, allowing for adaptation to the change in light availability which explains the peak expression of many miRNAs at this time. The circadian rhythm system is regulated by a number of different factors, including the release of hormones. In particular, potential target genes of differentially expressed miRNAs are abundantly enriched in the pathways involved in the endocrine system due to the physiological function of the eyestalk. The eyestalk is a crucial endocrine organ in crustaceans, regulating the secretion of various hormones, and contains a variety of precursors that can regulate, among others, melatonin, molt-inhibiting hormone, and hyperglycaemic hormone, hence affecting the long-term survival of crustaceans^56^. The secretion of a variety of important neuropeptides in the eyestalk, such as the crustacean hyperglycemic hormone (CHH), the molt-inhibiting hormone (MIH), the pigment dispersing hormone (PDH), and the red pigment-concentrating hormone (RPCH), is circadian in crustaceans^14^. Studies have shown that miRNAs have a regulatory effect on the secretion of these hormones, but whether miRNAs in crustaceans can directly exercise circadian regulation by regulating hormone secretion is unclear^57^. Our present miRNA profile indicates that crustaceans have the potential to regulate physiological functions in the circadian cycle by regulating certain hormones such as crustacean hyperglycemic hormone and pigment dispersion hormone through miRNA.

Our study suggests the differential expression of miRNAs under circadian rhythms may contribute to the diurnal differences in immune and endocrine function in crabs. The up-regulated expression of some miRNAs in the evening may be related to their immune or hormone secretion. Abundant target genes are involved in the circadian-related pathway and enriched on circadian-control genes. Advanced research is necessary to clarify the mechanisms underlying the role of miRNAs in crustacean circadian rhythms, as there is currently little information in this field. There are still limitations in the high-quality data available on crustacean genomes, and crustacean miRNAs are scarce in some miRNA databases. To the best of our knowledge, this study is the first systematic, high-throughput sequencing analysis of miRNAs in *E. sinensis* eyestalks. Our work provides valuable insight into the mechanism underlying miRNA regulation of crustacean circadian rhythms and establishes an essential foundation for future research.

## Supplementary Information

Supplementary Figure S1.

Supplementary Figure S2.

Supplementary Figure S3.

Supplementary Figure S4.

Supplementary Legends.

Supplementary Table S1.

Supplementary Table S2.

Supplementary Table S3.

Supplementary Table S4.

Supplementary Table S5.

## Data Availability

The raw reads have been deposited into the NCBI database (Accession number SRR15367803).
