# Supplementary figures and images for: Deep sequencing of microRNAs reveals circadian-dependent microRNA expression in the eyestalks of the Chinese mitten crab Eriocheir sinensis
Source: Sci Rep. 2023 Mar 31;13:5253. doi: 10.1038/s41598-023-32277-1 (PMC10066325; doi:10.1038/s41598-023-32277-1)

# Length Distribution

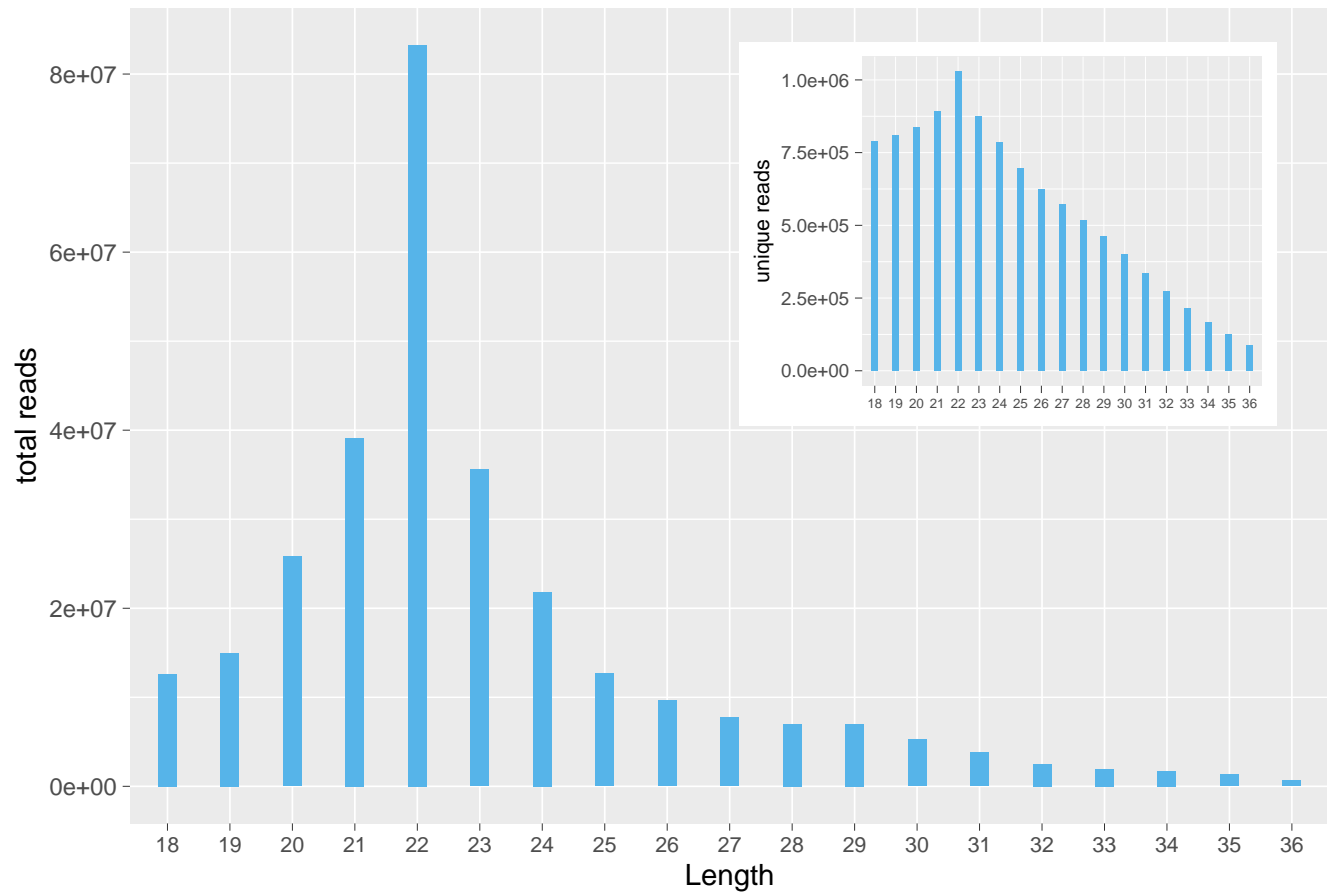

Supplement: Supplementary file 1 — Supplementary Figure S1. [file 41598_2023_32277_MOESM1_ESM.pdf]

a

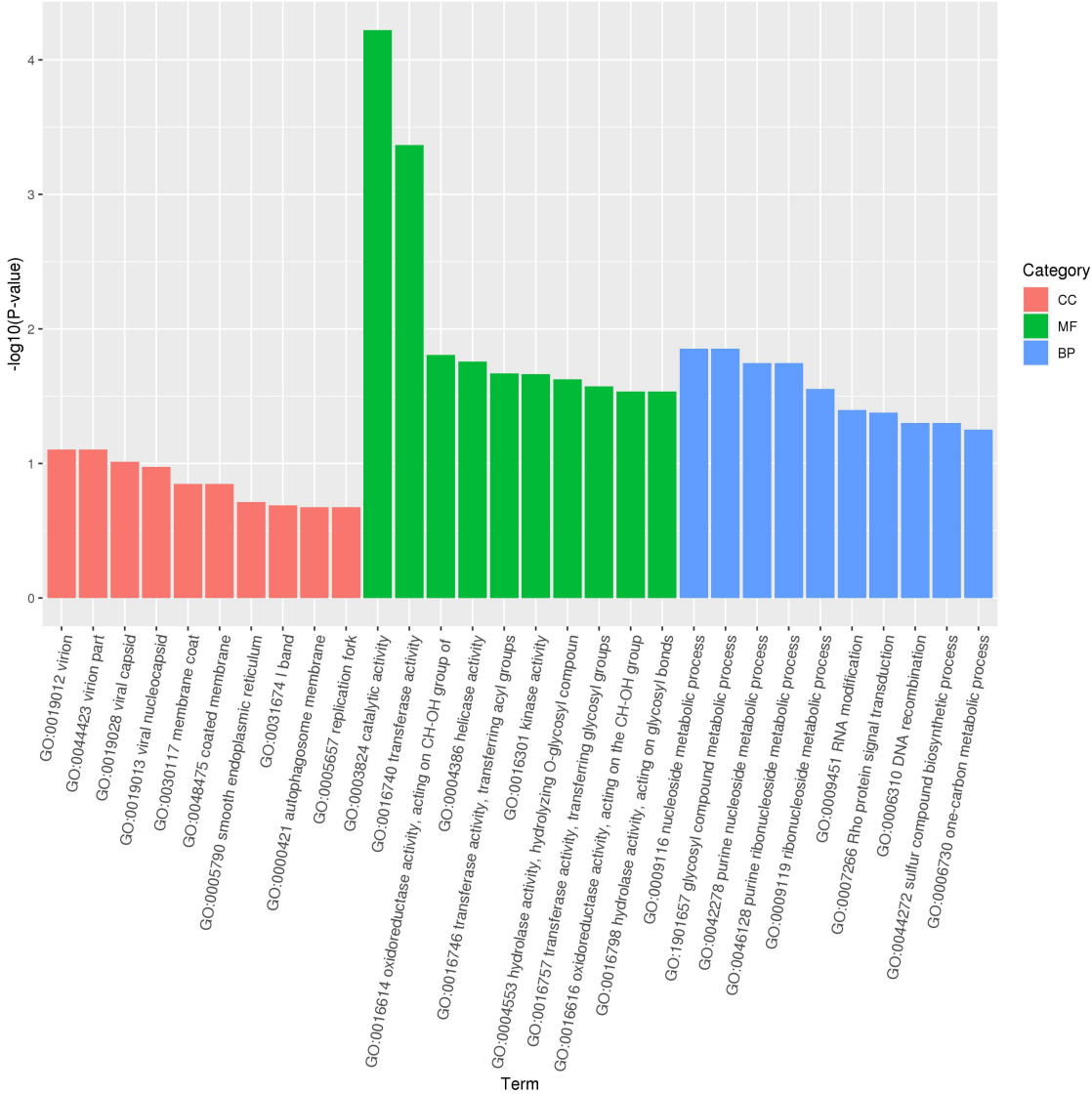

b

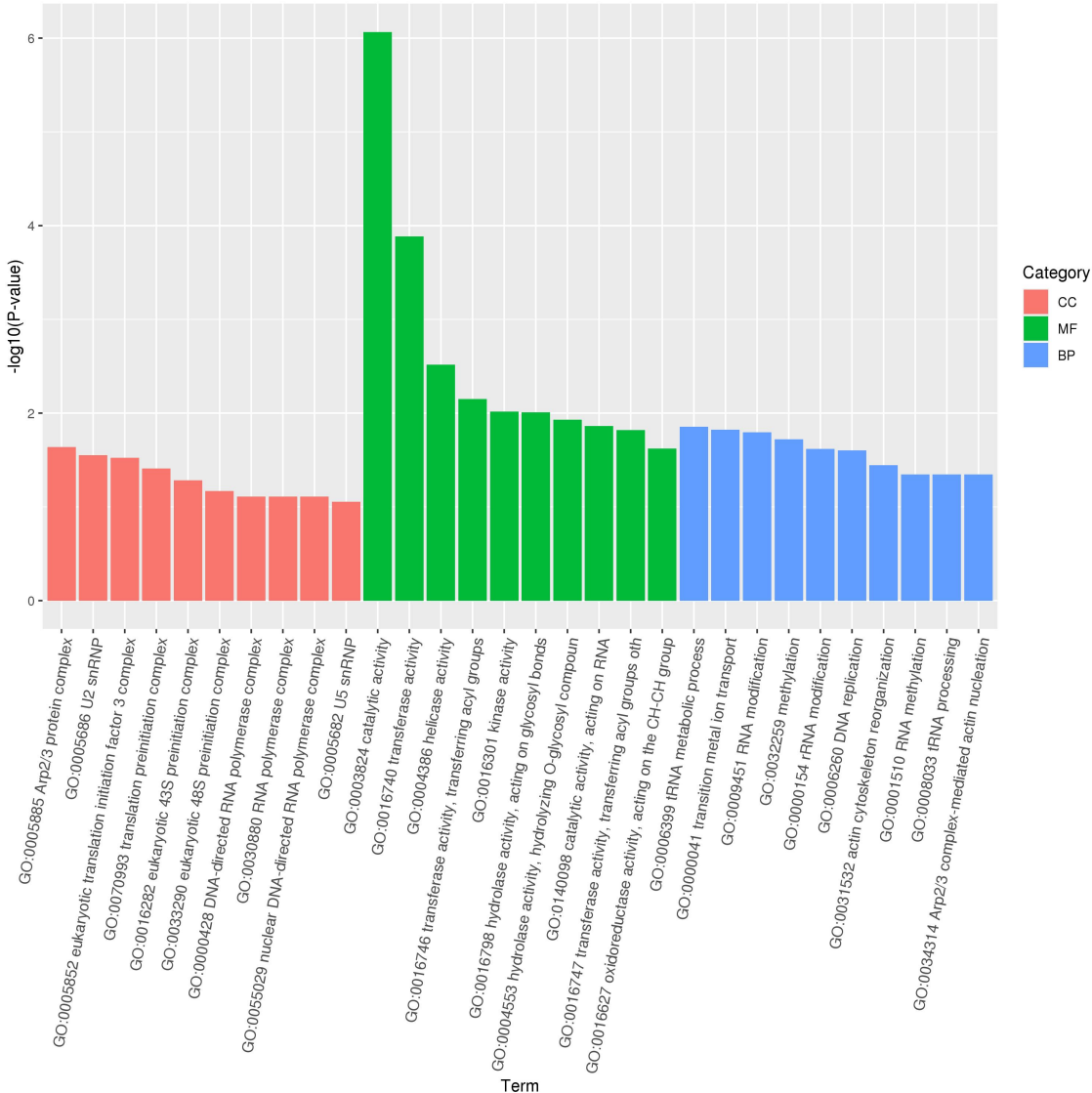

c

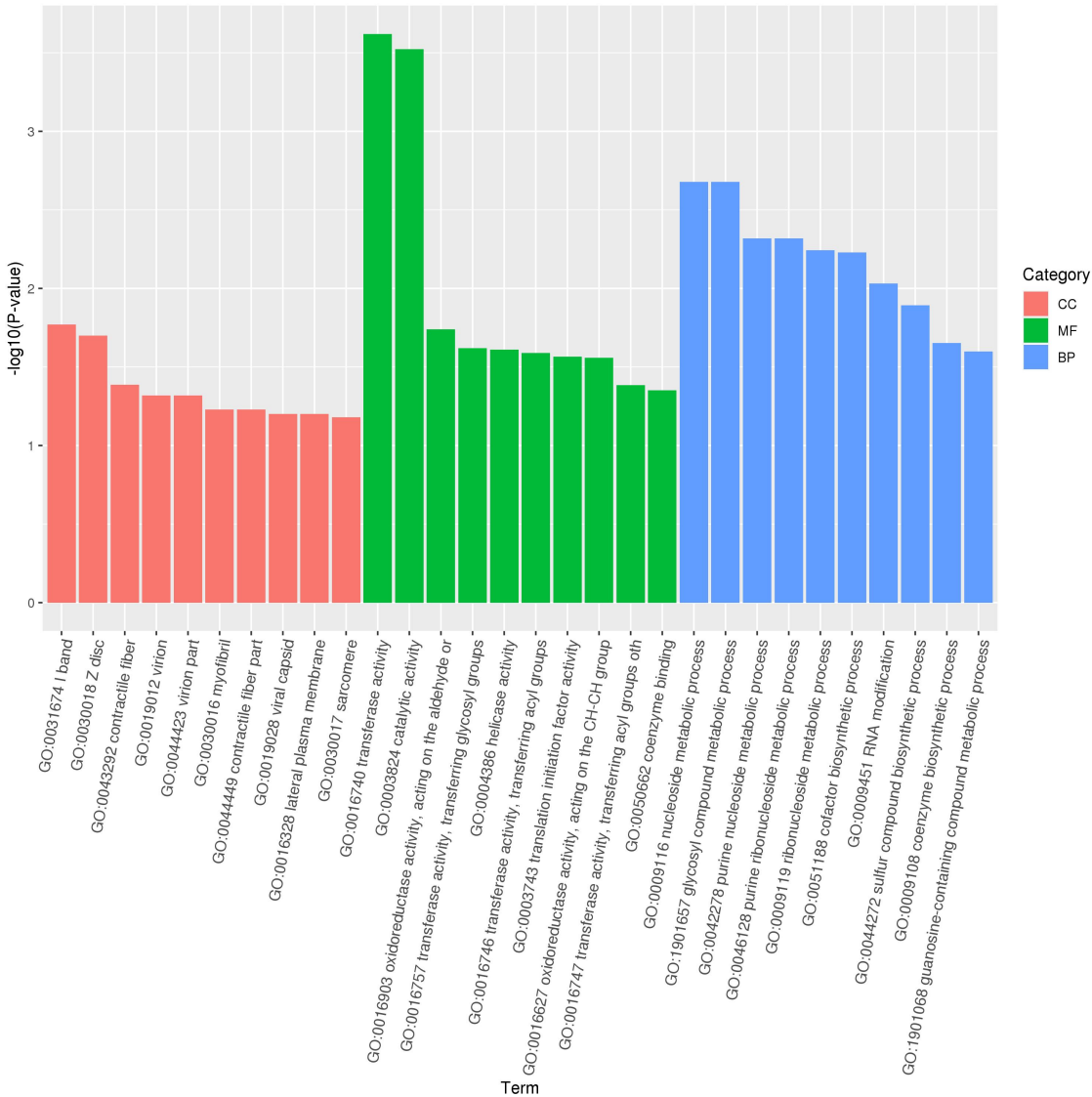

Supplement: Supplementary file 2 — Supplementary Figure S2. [file 41598_2023_32277_MOESM2_ESM.pdf]

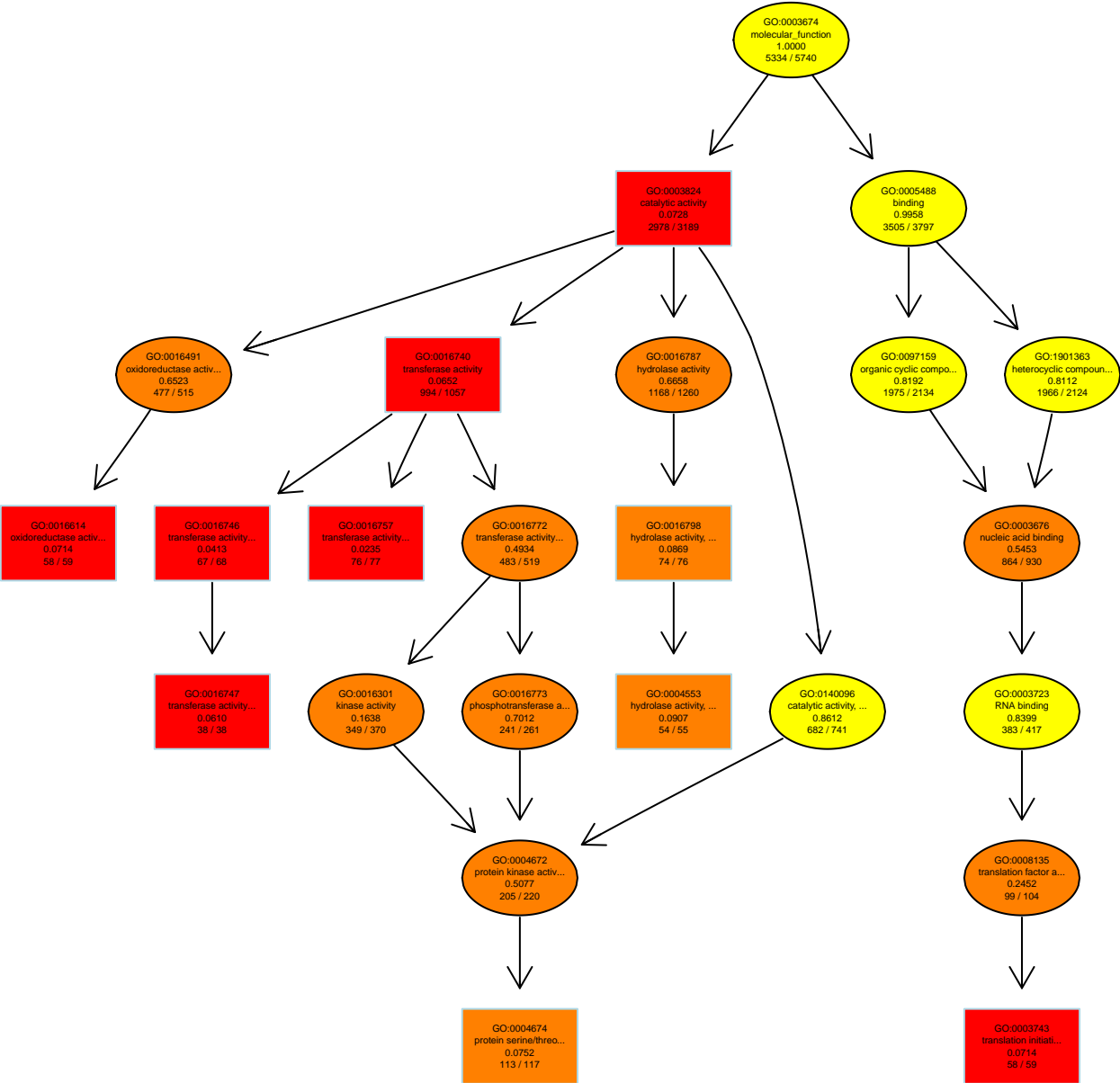

Supplement: Supplementary file 3 — Supplementary Figure S3. [file 41598_2023_32277_MOESM3_ESM.pdf]

# CIRCADIAN RHYTHM

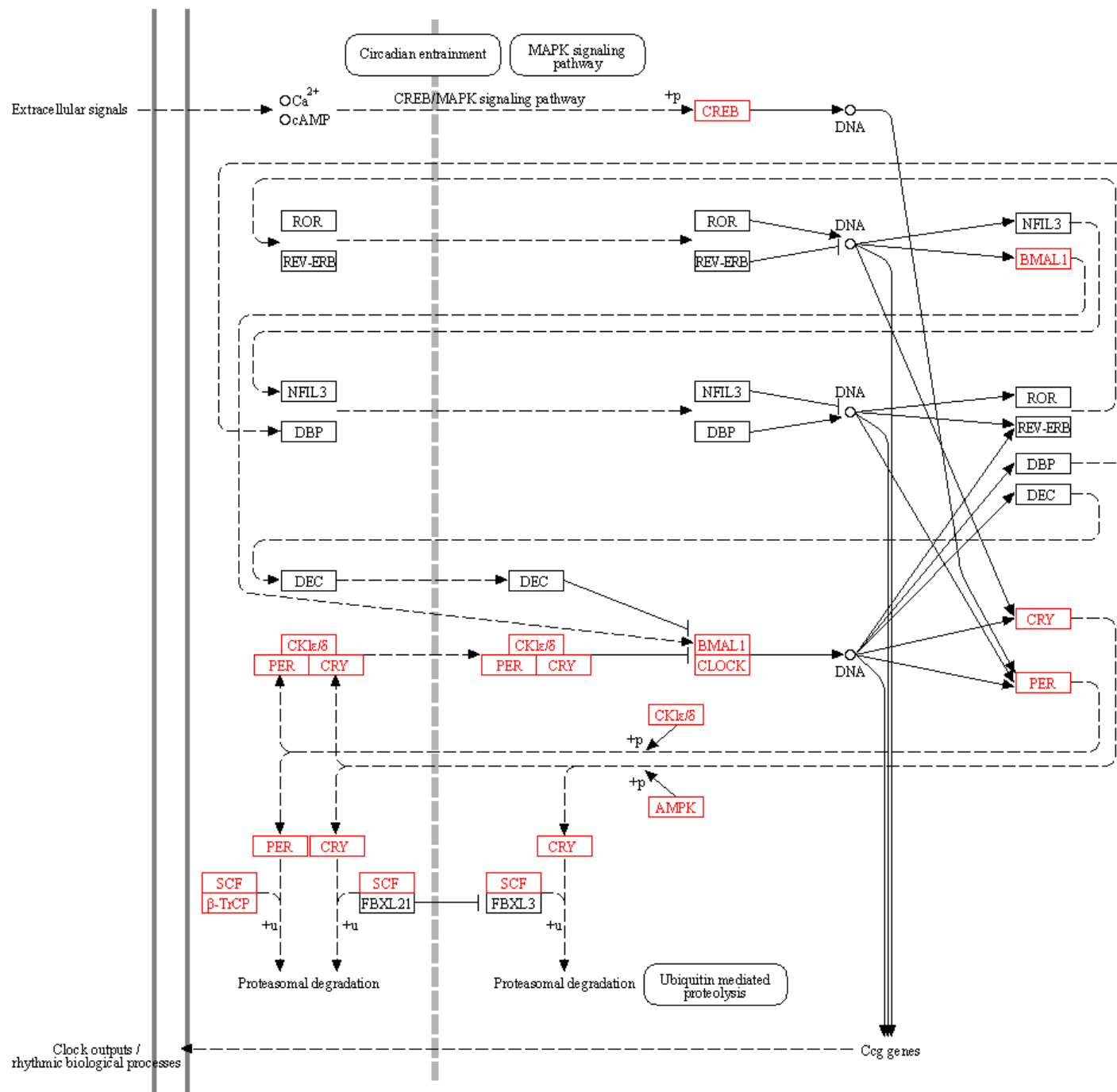

Supplement: Supplementary file 4 — Supplementary Figure S4. [file 41598_2023_32277_MOESM4_ESM.pdf]
