# Supplementary material for: Deep sequencing of microRNAs reveals circadian-dependent microRNA expression in the eyestalks of the Chinese mitten crab Eriocheir sinensis
Source: Sci Rep. 2023 Mar 31;13:5253. doi: 10.1038/s41598-023-32277-1 (PMC10066325; doi:10.1038/s41598-023-32277-1)
Supplement: Supplementary file 5 — Supplementary Legends. [file 41598_2023_32277_MOESM5_ESM.docx]

Figure S1. Length distribution and percentage of total reads and unique reads in small RNAs.

Figure S2. GO enrichment analysis of putative target genes for differentially expressed miRNAs. (a) The GO map of putative target genes in group 6:00 vs. 18:00. (b) The GO map of putative target genes in the 12:00 vs. 18:00 group. (c) The GO map of putative target genes in the 18:00 vs. 24:00 group.

Figure S3. KEGG enrichment analysis of putative target genes in circadian rhythm pathway. The nodes marked in red are enriched putative targeted genes of miRNAs.

Figure S4. KEGG map of Circadian Rhythm pathway. Pathway ID: Ko04710, KEGG imagery source: http://www.kegg.jp/kegg/kegg1.html (Red : putative target genes with differentially expressed miRNAs enriched to that gene).
